# Effect of Dry Heating on Some Physicochemical Properties of Protein-Coated High Amylose and Waxy Corn Starch

**DOI:** 10.3390/foods12061350

**Published:** 2023-03-22

**Authors:** Lili Mao, Pranita Mhaske, Asgar Farahnaky, Mahsa Majzoobi

**Affiliations:** School of Science, RMIT University, Bundoora West Campus, Plenty Road, Melbourne, VIC 3083, Australia

**Keywords:** physical modification, thermal treatment, protein coated starch, amylose, amylopectin

## Abstract

The dry heat treatment (DHT) of starch and hydrocolloid mixtures is gaining acknowledgement since hydrocolloids can enhance the efficiency of DHT. However, the DHT of a starch–protein mixture has been less investigated. In this study, the effects of different proteins including sodium caseinate (SC), gelatin, and whey protein isolate (WPI) added to high amylose and waxy corn starches (HACS and WCS, respectively) prepared by the dry mixing and wet method before and after DHT were studied. The DHT of both starches with WPI and SC prepared by the wet method increased the peak viscosity, but no change was observed when gelatin was added. Dry mixing of HACS with the proteins did not affect the peak viscosity before and after DHT. The gelatinization temperatures and enthalpy of both starches showed a slight decrease with the addition of all proteins and reduced further after DHT. The firmness, gumminess, and cohesiveness of the samples decreased upon DHT. The SEM results revealed that the granules were coated by proteins and formed clusters. Particle size analysis showed an increase in the particle size with the addition of proteins, which reduced after DHT. Under the conditions used, the wet method was more successful than dry mixing and the effects of WPI > SC > gelatin in enhancing the physicochemical properties of the tested starches after DHT.

## 1. Introduction

Starch is broadly used in industrial and food applications as a gelling agent, thickener, bulking agent, hygroscopic agent, colloidal stabilizer, and adhesive material. With the increase in industrial demand, there is a constant need to explore alternative and new sources of starch [1]. For many applications, native starch has some limitations including low interactions with water at temperatures below the gelatinization point, low shear stability, and a high tendency to retrogradation. To overcome these inherent drawbacks, native starch is often modified to meet the desired applications [2]. Starch modification can be designed to reduce gel syneresis, paste gelling tendencies, and retrogradation, while improving the gel and paste texture, adhesion, film formation, paste clarity, and sheen [3,4].

Heat treatment is one of the physical methods of starch modification that is gaining popularity in the food and pharmaceutical industries as it is an affordable, easy, safe, and green (chemical-free) method of starch modification [5].

Dry heat treatment (DHT) involves the heating of starches with low moisture content (less than 10%) at 120–130 °C for 2–4 h [6]. DHT can markedly alter the ratio of amylose to amylopectin, the starch molar mass, the hierarchical structure, and the functional properties of starch, resulting in comparable effects such as those induced by chemical crosslinking [7,8,9]. Some studies have shown a decrease in starch digestibility since DHT can limit the accessibility of amylase to starch macromolecules [9]. The water solubility of starch is enhanced while the swelling power is reduced by DHT [9,10]. DHT starch is considered as a chemical-free counterpart of chemically cross-linked starch and can be used in food products to support stable viscosity (e.g., jam, puddings, soups), low GI foods such as bread and noodles as well as the production of biodegradable films [9,11,12]. 

It has been reported that the presence of other hydrocolloids such as proteins and polysaccharides can enhance the cross-linking effects of DHT on starch [2,3,13,14,15]. During the process of dry heating, the hydrocolloids added to starch can act as crosslinking agents capable of forming graft copolymers through ester formation, enhancing the textural properties and stability of starch pastes and gels [13,16]. Compared to chemical modification methods, DHT has the advantage of reducing the processing time and processing costs and eliminating chemicals [12]. Due to the importance of proteins in food products and the co-existence of protein and starch in many foods, studying the effects of DHT on mixtures of starch and proteins is of great interest. It has been indicated that proteins can form complexes with starch and exhibit some unique functional and textural properties [17,18]. Asghari et al. [8] reported that the particle size of rice starch granules increased after heat-treating the starch with egg white protein, while synergy between the starch and protein was evident. Zhu et al. [13] reported that the DHT of rice starch in the presence of whey protein isolate resulted in the aggregation of the starch granules, reduction in the gelatinization temperature, destruction of the long- and short-range molecular orders of starch, and a significant increase in the oil-binding capacity of the starch. It has been found that the DHT of starch with a soy protein isolate (SPI) had no significant effect on the type and degree of crystallinity of starch, but could significantly enhance the storage and loss modulus of its gel, which is related to the crosslinking effects of SPI on the starch molecules [18,19]. Although the effects of proteins on the functional properties of starch under DHT are documented, comparative studies on the different types and concentrations of proteins and starches and the optimum processing conditions have been less investigated.

Whey protein isolate (WPI), sodium caseinate (SC), and bovine gelatin (G) are commonly used in the food industry due to their availability, cost-effectiveness, nutritional value, and diverse functional properties [20,21]. Therefore, in the present study, the effects of these proteins on the properties of waxy corn starch (WCS) and high amylose corn starch (HACS) were investigated with and without dry heat treatment. In addition, to make the process more environmentally friendly, dry mixing of the protein and starch (as a novel approach) was also compared with the wet preparation method (as a common practice). The dry mixing of the samples can reduce the amount of water and energy used for starch modification. However, the effectiveness of the dry mix procedure has been less studied, which was investigated in this research. 

## 2. Materials and Methods

### 2.1. Materials

High amylose corn starch (HACS, amylose content: 76%) and the unmodified waxy corn starch (WCS) of pure amylopectin with only a traceable amount of amylose were purchased from Sigma-Aldrich Pty. Ltd. (Sydney, NSW, Australia). Gelatin with a bloom number of 125 and SC with a protein content of 91% was also obtained from Sigma-Aldrich Pty. Ltd. (Sydney, NSW, Australia). WPI (protein content: 94%) was secured from Fonterra (Auckland, New Zealand).

### 2.2. Methods

#### 2.2.1. Sample Preparation

To study the effects of starch type (waxy and high amylose), selected proteins (WPI, SC, and gelatin), DHT, and preparation methods (wet or dry mixing of the starch and protein), the following samples were prepared according to Lim et al. [19] with some modifications. 

##### Wet Preparation of the Starch-Protein Mixtures without DHT

Protein solutions were prepared separately by adding 3.7 g (dry basis) of WPI, SC, or gelatin to approximately 95 mL MilliQ water (MilliQ Reference A+ system, from MERCK MILLIPORE, Germany) and stirred continuously on a magnetic stirrer at 65 °C for gelatin and at room temperature (21 °C) for SC and WPI until complete dissolution. The gelatin solution was left at room temperature to cool down. Then, 120 g (dry basis) of either HACS or WCS was dispersed into the prepared protein solutions, respectively, to achieve a starch:protein weight ratio of 97:3 while the starch + protein to water weight ratio was maintained at 4:3. The dispersions in a closed container were stirred for 60 min at room temperature using a magnetic stirrer. These were then transferred to Petri dishes and dried in a conventional oven at 45 °C until the moisture content was less than 10%. A drying temperature of 45 °C was selected to ensure that the starch granules and proteins retained their native structure. The drying step may cause some unintentional annealing of the starch samples. The samples were then cooled down to room temperature, ground using a coffee grinder, and sifted through a 200 μm sieve, packed in glass jars, and stored in a desiccator at room temperature for further experiments. The samples prepared with the wet method before DHT are referred to as BHT (before heat treatment), followed by the combination of protein and starch used (i.e., BHT WPI/SC/G-HACS/WCS).

##### Wet Preparation of the Starch–Protein Mixtures with DHT

The powders obtained in the previous step were used for DHT by heating them in a conventional air-forced oven at 120 °C for 4 h [19]. The samples were then cooled down to room temperature, milled, and sifted to the particle size of 200 μm, packed in glass jars, and stored in a desiccator at room temperature. The samples prepared with the wet method have the prefix AHT (after heat treatment), followed by the combination of protein and starch used (i.e., AHT WPI/SC/G-HACS/WCS).

##### Dry Preparation of the Samples

Samples of starch and proteins with the same concentrations above-mentioned were mixed in dry form (without solubilizing in water) as follows: Dry mix—(WPI/SC/G—HACS/WCS) BHT by the dry mixing of native starches and proteins (no heat treatment); and Dry mix—(WPI/SC/G—HACS/WCS)-AHT where the dry mixtures of starch and proteins were heated at 120 °C for 4 h.

#### 2.2.2. Pasting Properties

The pasting properties of the controls and the modified starches were determined in triplicate using a Rapid Visco Analyzer (RVA) (Model 4500, Perten Instrument, Sydney, NSW, Australia) following the method described by Qiu et al. [18] with slight modifications. A total of 3.5 g of starch (dry basis) was directly weighed into the RVA canister. To have the same starch water concentrations for all RVA experiments, based on starch with 10% moisture, the calculated amount of MilliQ water was added to the canister for all samples.

The samples were mixed vigorously a” 960’rpm for the first 10 s to disperse the starch granules. After mixing, the paddle speed was lowered to 160 rpm and kept constant throughout the test. Samples were heated from 21 °C to 50 °C and equilibrated for 1 min before ramping up the temperature to 95 °C. The samples were held at 95 °C for 2 min 30 s before being cooled down to 50 °C, and finally held at 50 °C for 2 min. A ramp rate of 12 °C/min was used throughout. Parameters including pasting temperature, peak viscosity, trough viscosity, final viscosity, breakdown (peak viscosity—trough viscosity), and setback (final viscosity—trough viscosity) were recorded.

#### 2.2.3. Thermal Properties

The thermal properties of all samples were determined following the method outlined by Majzoobi, Saberi, Farahnaky, and Tongdang [22] using a differential scanning calorimeter (DSC Q2000, TA Instrument, Sydney, NSW, Australia). A 5 mg sample (dry weight basis) was accurately weighed into an aluminum pan and an equal amount of MilliQ water was added to achieve a water–sample ratio of 1:1. Afterward, the pan was hermetically sealed and allowed to stand for 24 h at 21 °C for complete rehydration. All the pans were loaded into the DSC followed by the pre-setup scanning temperature and heating profile. Samples were heated at a rate of 10 °C/min within a temperature range of 10–100 °C using an empty aluminum pan as a reference. Onset temperature (T_o_), peak temperature (T_p_), conclusion temperature (T_c_), and enthalpy of gelatinization (ΔH) were calculated directly from the DSC curves.

#### 2.2.4. Textural Properties of Starch Gels

Starch gels were prepared by directly pouring the hot starch paste obtained at the end of the RVA run into a 10 mm deep plastic cylindrical mold with a 10 mm diameter. To avoid moisture loss, the molds were covered with parafilm and stored at 4 °C overnight. This ensured uniformity in the cooling and gelation conditions for all samples prior to further experimentation.

Textural profile analysis of the prepared starch gels was carried out using a Texture Analyzer (TA.XT. Plus, Stable Microsystems Ltd., Godalming, UK) equipped with a 25 mm diameter cylindrical aluminum probe. The samples were compressed to 30% of their original height using a double compression test under the following conditions: 3.0 g trigger force, compression speed of 0.25 mm/s, and an interval of 10 s between the two cycles. The TPA parameters measured were based on the compression force versus time curves. Gel firmness was determined as the maximum force during the first compression (height of the first peak), the cohesiveness was calculated as the ratio of the area under the second peak (A2) to the area under the first peak (A1), gumminess was calculated by multiplying the value of hardness and cohesiveness, and elasticity (springiness) was determined as the ratio between the recovered height after the first compression and the original gel height [23].

#### 2.2.5. Particle Size Distribution

The particle size distribution was determined by a Malvern Mastersizer 3000 Laser diffraction Aero system (Malvern Instruments Ltd., Malvern, UK). The 100 mg starch samples were weighed and swiftly transferred into the sample cell of the particle size analyzer. The dispersion conditions including 2 bar air pressure and 60 % feed rate were maintained for all samples. The refractive index used was 1.5. Each sample was analyzed in triplicate [24].

#### 2.2.6. Polarized Light Microscopy

Suspensions of all samples were prepared using a 4:1 water–sample ratio. A drop of the suspension was added onto a glass slide and covered with a coverslip before observations under a polarized light microscope (Eclipse 80i, Nikon, New York, NY, USA) at 50× magnification [24]. 

#### 2.2.7. Scanning Electron Microscopy (SEM)

The morphological characteristics of all samples were observed using a scanning electron microscope (Quanta 200, FEI, OR, Salem, MA, USA). A dry, finely ground sample was placed on 12.6 mm carbon-coated aluminum pans and coated with a thin film of iridium. Surface micrographs were captured in high vacuum mode with an accelerated electron beam of 25 kV, spot size of 4, and 1000× magnification [24].

#### 2.2.8. Statistical Analysis

All experiments were performed in triplicate. The experimental data presented in the tables were the average of the triplicate value ± standard deviation. Analysis of variance (ANOVA) was conducted while Tukey’s Honest Significant Difference tests were applied to determine the significant differences (*p* < 0.05, 95% confidence). Statistical Package for Social Science (SPSS, IBM, Albany, NY, USA) v 17.0 was used for conducting statistical analysis.

## 3. Results and Discussion

### 3.1. Pasting Properties

**Figure 1:**
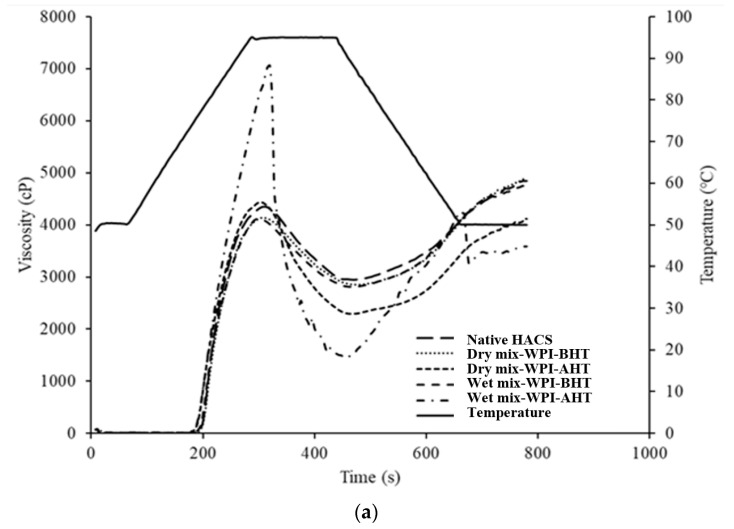

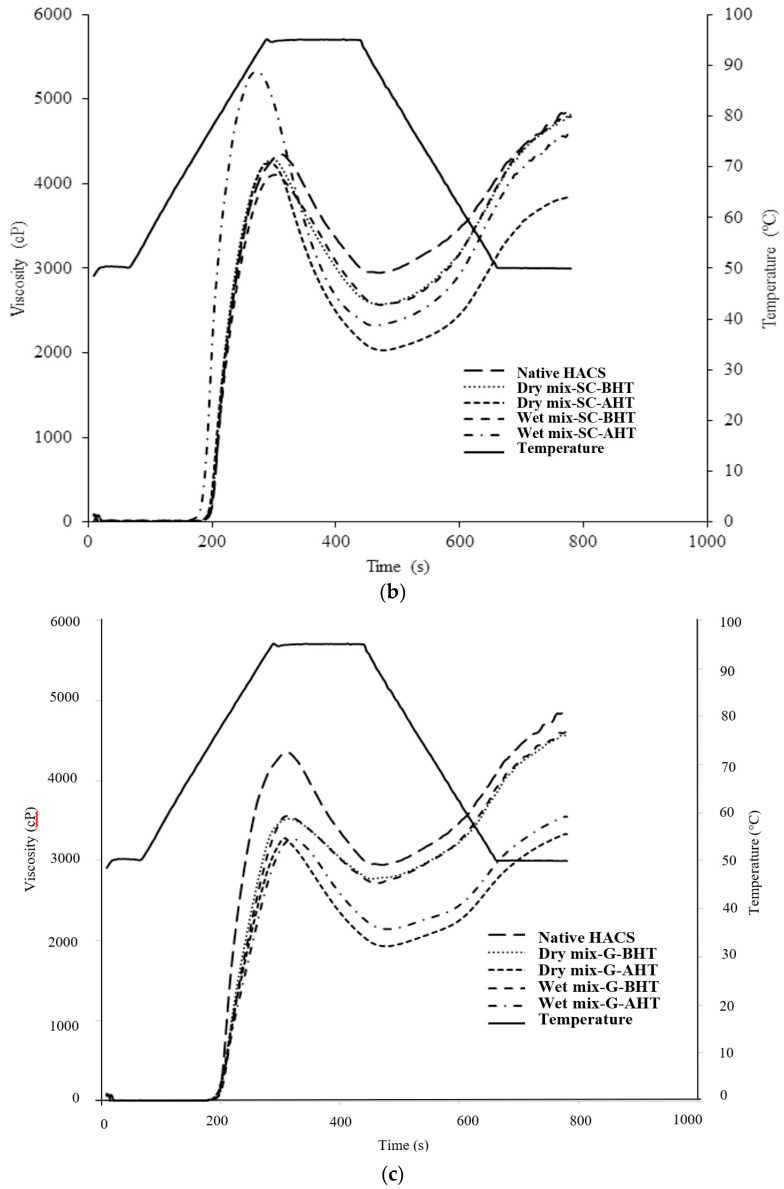

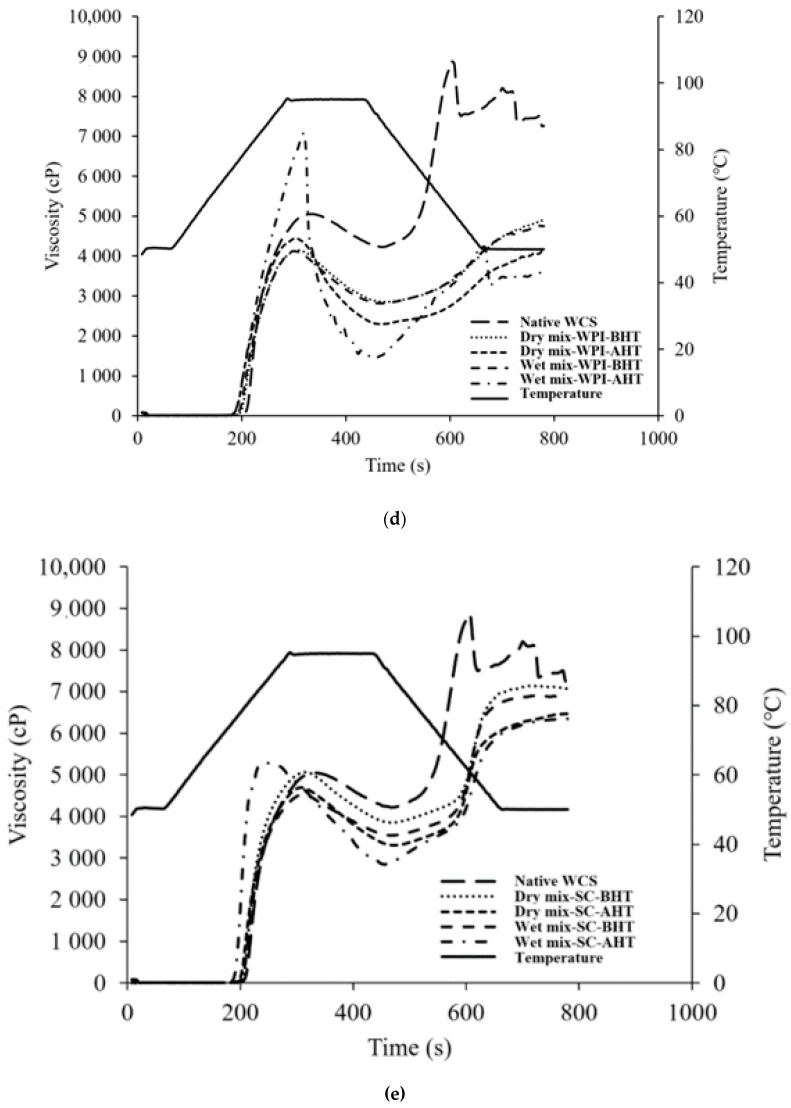

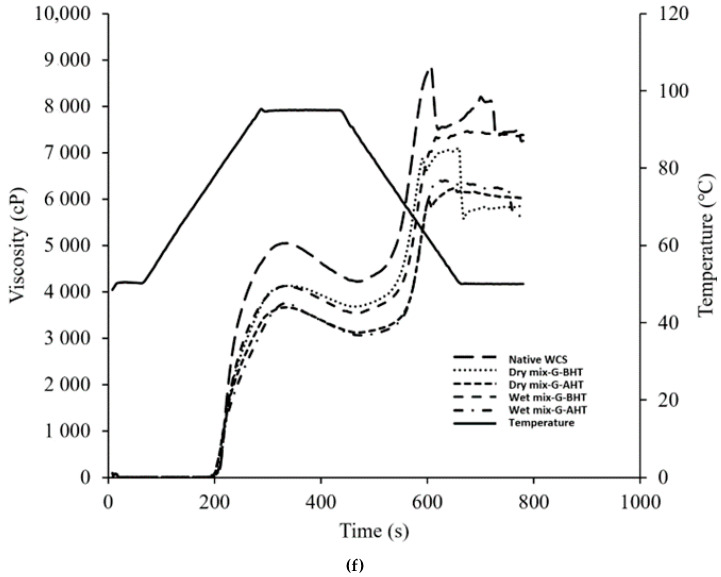
Pasting properties of native high amylose corn starch (HACS) (**a**–**c**), and waxy corn starch (WCS) (**d**–**f**), mixed with proteins (WPI—(**a**,**d**); SC—(**b**,**e**); G—(**c**,**f**)). BHT = before heat treatment; AHT = after heat treatment.

**Figure 2:**
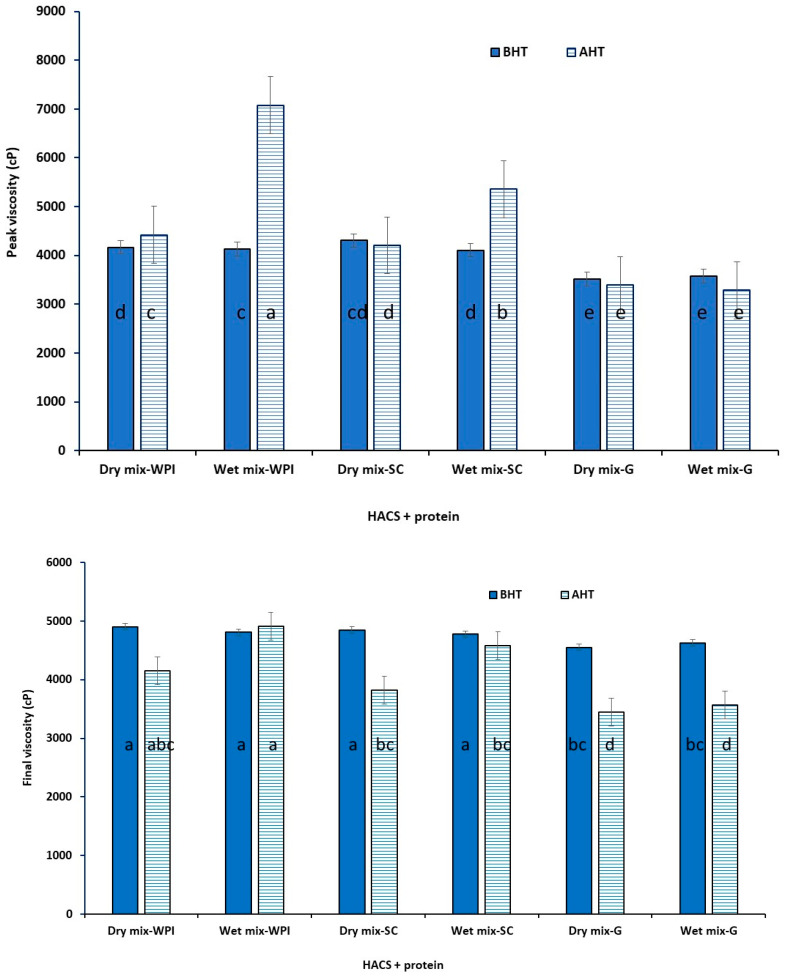
Peak and final viscosity of high amylose corn starch (HACS) and the protein mixtures before heat treatment (BHT) and after heat treatment (AHT). The different letters on the columns indicate statistically significant differences (*p* < 0.05).

**Figure 3:**
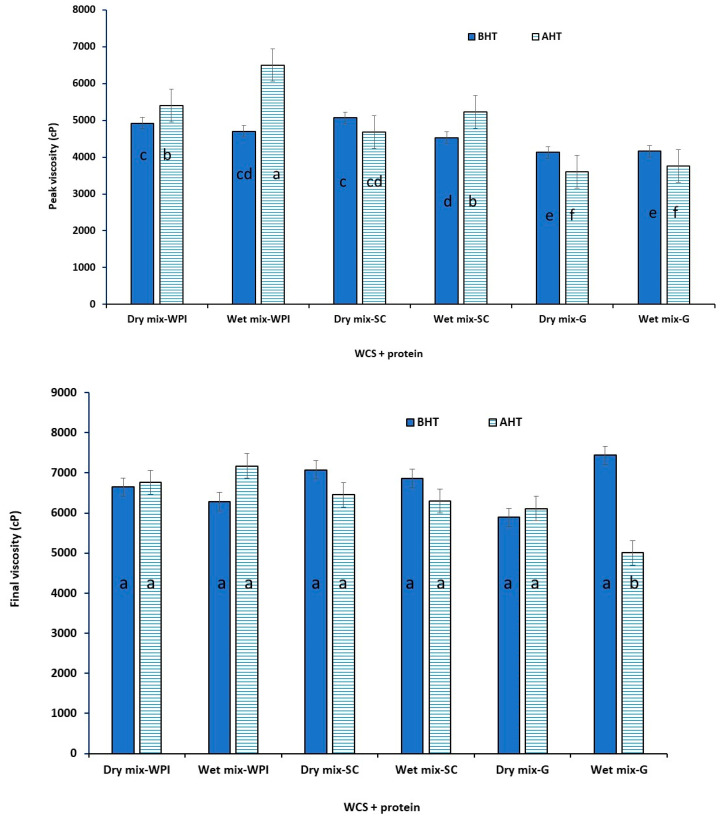
Peak and final viscosity of waxy corn starch (WCS) and protein mixtures before heat treatment (BHT) and after heat treatment (AHT). The different letters on the columns indicate statistically significant differences (*p* < 0.05).

Figure 1 shows the RVA graphs of all samples, and the peak, and final viscosities (as the two most important pasting properties) are presented in Figure 2 and Figure 3. Peak viscosity is the maximum viscosity during heating and is an indication of the granules’ ability to absorb water and swell. Final viscosity is related to the highest viscosity during the cooling cycle of the RVA and represents starch retrogradation during cooling [22]. 

It was found that the type of starch, type of proteins, and the preparation method (dry or wet) had significant effects on the pasting properties of the samples. For example, the mixing of HACS with the tested proteins had no effect on the peak viscosity before and after heat treatment, however, it reduced the final viscosity of all samples. This may indicate that the low concentrations of the tested proteins could not enhance the limited water uptake and swelling characteristics of the high amylose. For dry mixtures of WCS with proteins, WPI caused an increase while SC and gelatin reduced the peak viscosity after DHT. WPI and gelatin had no considerable effect while SC caused a reduction in the final viscosity of WCS.

For wet mixtures of HACS and proteins, the peak viscosity increased when WPI and SC were added while it remained unchanged when gelatin was included. Similar results were observed for the WCS and proteins. The final viscosity of the HACS remained unaffected by the DHT of the wet mixtures of WPI and SC, but it was reduced when gelatin was added. For wet mixtures of WCS and proteins, the final viscosity increased when WPI was added, but decreased when SC and gelatin were included. The observed changes could be due to the interactions between the starch and proteins, the denaturation or coagulation of proteins during DHT, the interactions with water as well as the molecular and structural changes of starch and the ratio of amylose to amylopectin. Comparing the values of peak viscosity for HACS and WCS, it seems that the interactions of WCS with WPI or SC were more significant compared with the interactions of HACS with the same proteins before dry heating. Similar results were observed by Zhu et al. [13] for DHT rice starch and WPI. A high content of amylopectin in WCS seems to be the main contributor to the increased viscosity due to increased crosslinking with proteins.

Protein structure and concentration have significant effects on the pasting properties of starch [25]. Kumar, Brennan, Brennan & Zheng [26] (2022) reported that below WPI’s critical concentration of gelation (~90 g/L at neutral pH in the absence of salt), the dimers, trimers, and tetramers formed by the free thiol groups of the denatured whey protein binding with sulfhydryl groups of other protein are not enough to have an impact on the overall viscosity. Wang et al. [25] reported a higher peak viscosity of rice starch when mixed with WPI, but a lower peak viscosity when mixed with casein during the RVA test. They indicated that WPI, as a globular protein, can accelerate starch swelling, resulting in a rapid increase in the paste viscosity. However, the interaction of starch and casein was the opposite as it resulted in delayed water uptake (lower peak viscosity).

### 3.2. Thermal Properties

The onset (T_o_), peak (T_p_), and conclusion (T_c_) gelatinization temperature and enthalpy of the two starches with and without added proteins, before and after DHT are reported in Table 1a,b. Gelatinization is the disruption of molecular order within a starch granule that results in irreversible changes in its properties such as swelling of the starch granule, loss of crystallinity and birefringence, and starch solubilization. Generally, high amylose starches show higher gelatinization temperatures and lower enthalpy than their waxy counterparts. The gelatinization temperature of HACS used in this research was slightly lower than the previously reported values. This can be related to the differences in the amylose content, molecular structure, starch purity, and experimental conditions [27]. The results showed that DHT induced significant changes in the thermal behavior of corn starch. The gelatinization temperatures of both starches showed a slight decrease with the addition of all proteins, which was reduced further after DHT. It is possible that the heat treatment induces the partial structural disintegration of starch granules that enables its melting at lower thermal energy. The drop in gelatinization temperatures corresponded with the decreasing trend of the pasting temperatures seen in the previous section. The variations in the gelatinization temperature can be due to the structural rearrangement of starch granules upon thermal treatment, which also complies with changes in the pasting properties. The gelatinization transition temperatures (onset and peak) are indicative of the crystallinity of the starches, with the more perfect crystallites translating to higher onset temperatures [28]. In the presence of added proteins, prior to heat treatment, ‘T_o_’ showed a slight increase, which dropped after heat treatment. Ma, Zhu, and Wang [29] speculated that an increase in the gelatinization temperatures could be a result of delayed gelatinization due to the reduced water available for the starch granules. Zhu et al. [13], while studying the effect of WPI on rice starch following DHT, commented that the hydrophobicity of the starch was improved due to the exposure of hydrophobic groups of the partially denatured WPI, which reduced the amount of water absorbed by the starch granules during gelatinization, leading to an increase in the gelatinization temperature. This was further translated into a drop in enthalpy caused by the disruption of amylose chain associations, leading to lower thermal energy for phase transformation, although the degree of each effect depended on the specific protein used. Sun, Gong, and Xiong [30] suggested that the DHT induced structural changes in the amorphous regions of the starch granules, thereby decreasing the onset temperature. Similar findings were reported in cassava starch with CMC and sodium alginate addition as well as corn starch and waxy corn starch with SPI [3,18,31]. 

### 3.3. Textural Properties

The textural properties of native starch gels and those mixed with selected proteins before and after dry heating are summarized in Table 2a,b. It can be seen that the gel firmness decreased with just the dry mixing of protein with the starches for both the starches and all proteins. A similar trend was observed for gumminess and cohesiveness in the dry mixed samples. There was no change to the springiness in all samples. The addition of gelatin appears to have had the maximum impact on both starches, especially WCS with firmness, cohesiveness, and gumminess dropping to 50, 75, and 33% of native starch. A relatively smaller drop was seen in the cohesiveness in HACS on dry mixing with the proteins. Similar findings were observed for wheat starch with the addition of soy protein isolate (SPI) [32]. They suggested that the hardness of starch gels significantly decreased with the addition of SPI due to its interaction with amylose, exposing branched amylopectin through hydrogen bonds, which imparts the softness and weakness of the gel matrix.

Dry heating reduced the firmness of the gel by 25, 24, 19, 10, and 16% in HACS-WPI, HACS-SC, HACS-G, WCS-WPI, and WCS-SC, respectively. WCS-G, however, showed a 35% increase in the gel firmness. The cohesiveness of the starches with added WPI or SC remained fairly constant after dry heating. HACS-G showed a slight drop while WCS-G showed a 14% increase in cohesiveness after dry heating. The drop in cohesiveness indicates the disruption of the internal bonds in starch granules caused by heat treatment. The WCS-G bond, however, seems to strengthen during the heat treatment. The gumminess for all samples dropped after DHT, except for WCS-G, which showed a 60% increase.

### 3.4. Particle Size Distribution

A bimodal particle size distribution was obtained for both HA and WCS (Supplementary Data Figure S1). The changes in the particle size distributions of starch as a result of added proteins and DHT are summarized in Table 3. It was found that the average diameter of all the protein-bound starch granules was higher than that of the native starches. The average diameter of the particles ranged between 30 and 80 µm, while the individual particle sizes ranged between 9 and 250 µm. This wide range of particle size was observed due to the partial clumping of starch granules, resulting in a mixture comprising of free individual starch granules as well as those bound together, as seen in the SEM micrographs. For the SC and gelatin-treated starches, the average cluster size increased by three times in HACS and by five and three times, respectively, in WCS. All of these starches showed a decrease in particle size after DHT due to the compaction of the individual starch granules within a cluster. This restricted the swelling of the starch granules after DHT translates into the drop in final viscosity, as seen in the corresponding gels in Section 3.1. Starches with added WPI also showed a 2-fold increase in the average particle diameter. Interestingly, however, after DHT, the particle size increased 1.5 times for WPI-HACS and 2-fold for WPI-WCS. This is also in alignment with the increase in the final viscosity of the gels, as seen in the RVA results. Gulzar, Bouhallab, Jeantet, Schuck, and Crouguennec [33] suggested that heat-induced denaturation of WPI in the dry state led to aggregation formed with disulfide bonds and covalent crosslinks. Zhu et al. [13] showed that the DHT of rice starch with added WPI led to the formation of larger, tighter, and more stable aggregates due to the aggregation of WPI and crosslinking between WPI and starch. They also reported that increasing the dry heating time led to an increase in the size of the starch aggregates.

The SEM results discussed in the next section also back the observations thus far. Li et al. [14] noted that the dry heating of rice starch without the addition of gums had no remarkable effect on the particle size, nor did the starch-carboxymethyl cellulose mixture before DHT. After the heat treatment, however, a drastic increase in the particle size was observed.

### 3.5. Scanning Electron Microscopy (SEM)

**Figure 4:**
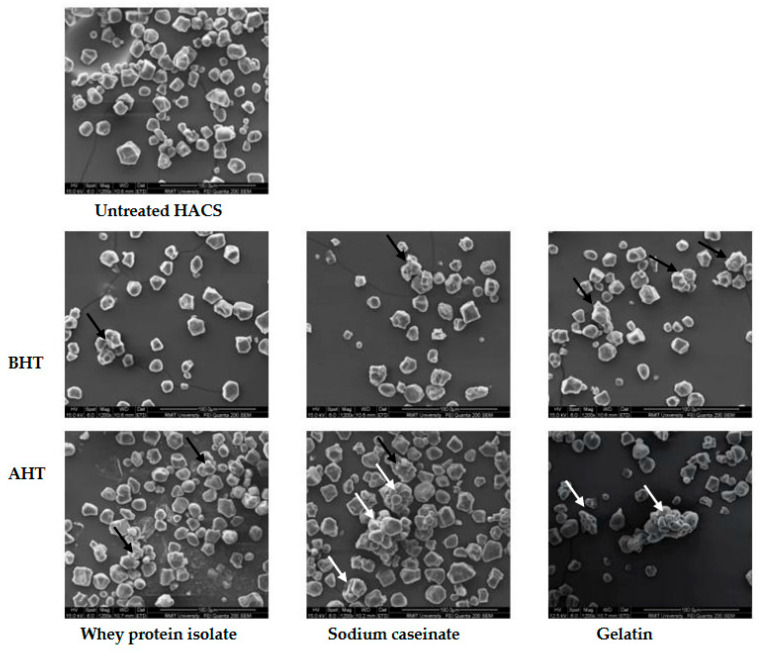
Scanning electron micrographs captured for high amylose corn starch (HACS) and in combination with proteins prepared by the wet mixing method before and after dry heat treatment under 1000× SEM. Arrows on the micrographs show that the protein coated the aggregated granules.

**Figure 5:**
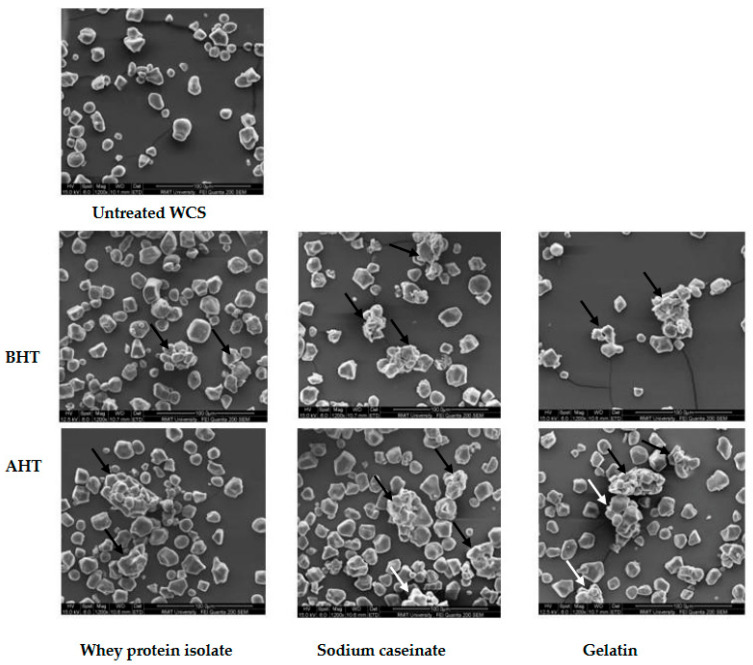
Scanning electron micrographs captured for the untreated waxy corn starch (WCS) and in combination with proteins prepared by the wet mixing method before and after dry heat treatment under 1000× SEM. Arrows on the micrographs show that the protein coated the aggregated granules.

Scanning electron micrographs of HACS and WCS with added proteins before and after DHT are depicted in Figure 4 and Figure 5. The untreated starch granules had typical spherical, angular, and polygonal shapes with smooth surfaces.

There was visible cluster formation with agglomeration in the treated starches, especially after DHT, as also seen in the works of Sun et al. [30] and Zhu et al. [13]. It has been found that WPI is aggregated during heat treatment and these aggregates form cross-linkages with starch granules and create a compact structure. DHT enhances WPI aggregates and their stability and interactions with starch granules, causing further changes to the starch properties [13]. Similar interactions may take place for CS and gelatin, leading to starch agglomeration, which requires further investigations.

As can be seen from the micrographs, however, not all of the starch granules were clumped, and a considerable amount still exists as individual particles, most likely caused by the disruption of the clusters during grinding. This explains the wide range of particle size distribution seen in the previous section. The proteins formed an evident coat on the starch granules, connecting at multiple points, which possibly restricts swelling, as mentioned in the previous sections. The surface of the starch granules, however, remained smooth and did not exhibit the formation of any fissures or amylose leaching, which have been reported in previous studies during DHT [34]. In addition to the macroscopic coating of protein on the starch granule surfaces as seen in the SEM micrographs, the protein was also adsorbed on the surface of the starch granules microscopically [13,35]. Noisuwan et al. [35] proved this while studying the effect of WPI on normal and waxy rice starch using SDS-PAGE and confocal laser scanning microscopy (CLSM). They even suggest that a certain amount of protein diffuses into the starch granules through surface holes and interior channels, although this has not yet been confirmed. Ryan and Brewer [36] suggest that the endogenous protein of the starch granules may mediate the adsorption of the exogenous protein.

### 3.6. Polarized Light Microscopy

**Figure 6:**
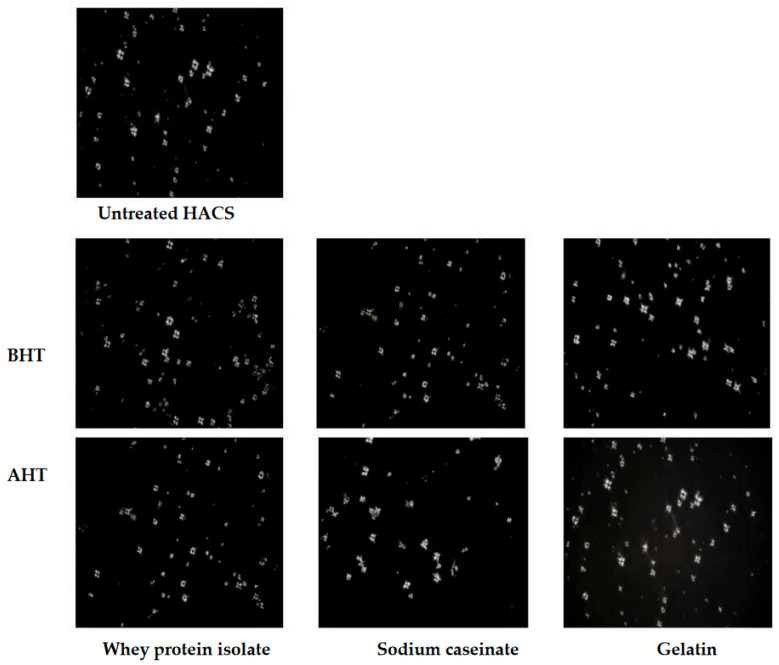
Light microscopy images captured for high amylose corn starch (HACS) and in combination with proteins prepared by the wet mixing method before and after dry heat treatment under a 50× lens.

**Figure 7:**
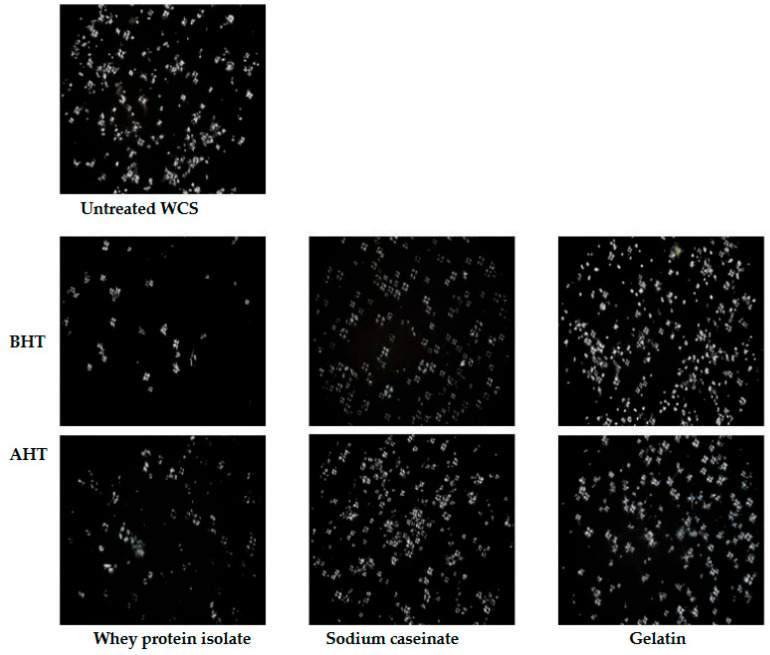
Light microscopy images captured for waxy corn starch (WCS) and in combination with the proteins prepared by the wet mixing method before and after dry heat treatment under a 50× lens.

Starch granules comprise crystalline and amorphous regions in which molecular chains are arranged in order and disorder, respectively. This leads to an optical anisotropy arising from the differences in density and refractive index, which leads to the formation of birefringence when polarized light passes through the starch granules [37]. The birefringence patterns of untreated HA and WCS, along with protein-modified starch before and after DHT are shown in Figure 6 and Figure 7. The untreated starches showed a distinct birefringence at the center of the native corn starch with a well-defined quadrant. This suggests that the crystallites in the unmodified samples were radially oriented [38]. When mixed with the proteins before and after DHT, there was no significant change in the birefringence, indicating that the addition of proteins and the heat treatment had little effect on the crystallinity of the starch granule. Numerous studies, however, have reported that prolonged heat treatment led to the formation of internal cavities in the granules, whilst the Maltese crosses still existed [39,40]. 

## 4. Conclusions

The present study documented the impact of DHT on HACS and WCS starches with added proteins. The modification resulted in desirable physiochemical changes such as increased peak viscosity, reduced enthalpy and gelatinization temperatures, and decreased gel firmness, gumminess, and cohesiveness, which could not be obtained by only mixing starch and the selected proteins. The addition of the proteins formed a thin coating on the starch granules, forming clusters that compacted in size during heat treatment, without affecting the crystallinity of the granules. The variations brought about in the properties were influenced by the type of protein used, the ratio of amylose–amylopectin of the native starch, and the mixing method (dry or wet mixing). It was found that the wet preparation method was more successful than the dry mixing method in enhancing the functional properties of the treated starches through the addition of low concentrations of the proteins. Amongst the proteins, WPI was the most effective and gelatin was the least effective protein on the functional properties of starch before and after DHT.

The positive outcomes of this study are encouraging since a small concentration of protein can boost the starch viscosity without using chemicals. With the further exploration of different protein–starch interactions, the results of this study can find widespread applications in the food and pharmaceutical industries. Further work is required to study the effects of dry heat treatment on the molecular structure of both starches and proteins.

## Figures and Tables

**Table 1:** (**a**) Thermal properties of corn starch with high amylose (HACS) control and protein-modified HACS before and after DHT. (**b**) Thermal properties of waxy corn starch (WCS) control and protein-modified WCS before and after DHT.

(a)				
Samples	T_o_ (°C)	T_p_ (°C)	T_c_ (°C)	ΔH (J/g)
HACS	63.36 ± 0.52 ^a,b,c^	69.42 ± 0.03 ^b^	74.82 ± 0.03 ^e^	6.62 ± 0.21 ^a,b,c^
Dry mix-WPI-BHT	64.42 ± 0.14 ^a^	70.84 ± 0.41 ^a^	75.64 ± 0.08 ^c,d^	5.43 ± 0.24 ^d,e^
Dry mix-WPI-AHT	63.14 ± 0.13 ^a,b,c^	70.23 ± 0.70 ^a^	77.05 ± 0.44 ^a,b^	5.52 ± 0.39 ^c,d,e^
Wet mix-WPI-BHT	64.35 ± 0.26 ^a^	68.97 ± 0.09 ^b,c^	75.45 ± 0.08 ^c,d,e^	5.27 ± 0.46 ^d,e^
Wet mix-WPI-AHT	60.94 ± 0.26 ^d^	67.55 ± 0.04 ^d^	74.48 ± 0.04 ^e^	5.68 ± 0.24 ^a,b,c,d^
Dry mix-SC-BHT	63.40 ± 0.18 ^a,b,c^	69.98 ± 0.53 ^a,b^	77.52 ± 0.62 ^a^	7.52 ± 0.28 ^a^
Dry mix-SC-AHT	63.50 ± 0.28 ^a,b,c^	69.80 ± 0.11 ^a,b^	76.78 ± 0.25 ^a,b,c^	7.66 ± 0.18 ^a^
Wet mix-SC-BHT	62.68 ± 0.82 ^a,b,c,d^	69.18 ± 0.66 ^b,c^	77.01 ± 0.19 ^a,b^	6.78 ± 0.00 ^a,b^
Wet mix-SC-AHT	62.00 ± 0.86 ^c,d^	67.85 ± 0.18 ^c,d^	76.05 ± 0.49 ^b,c,d^	6.66 ± 0.30 ^a,b,c^
Dry mix-G-BHT	64.71 ± 0.25 ^a^	71.00 ± 0.54 ^a^	77.98 ± 0.732 ^a^	6.78 ± 0.31 ^a,b^
Dry mix-G-AHT	64.10 ± 0.13 ^a,b^	70.82 ± 0.36 ^a^	77.61 ± 0.50 ^a^	5.01 ± 0.42 ^e^
Wet mix-G-BHT	62.20 ± 0.37 ^b,c,d^	69.38 ± 0.11 ^b^	75.45 ± 0.45 ^c,d,e^	6.92 ± 0.42 ^a,b^
Wet mix-G-AHT	61.98 ± 0.65 ^c,d^	67.52 ± 0.24 ^d^	74.93 ± 0.01 ^d,e^	5.98 ± 0.18 ^b,c,d^
(**b**)				
**Samples**	**T_o_ (°C)**	**T_p_ (°C)**	**T_c_ (°C)**	**ΔH (J/g)**
WCS	65.65 ± 0.15 ^a,b^	72.25 ± 0.03 ^a,b,c^	80.16 ± 0.31 ^b^	9.49 ± 0.22 ^a^
Dry mix-WPI-BHT	65.79 ± 0.04 ^a,b^	73.13 ± 0.08 ^a^	81.67 ± 0.09 ^a^	9.08 ± 0.24 ^a,b^
Dry mix-WPI-AHT	65.37 ± 0.21 ^a,b^	70.94 ± 0.35 ^e,f^	79.05 ± 0.31 ^b,c,d^	7.21 ± 0.31 ^c,d^
Wet mix-WPI-BHT	65.97 ± 0.11 ^a,b^	71.98 ± 0.06 ^b,c^	79.50 ± 0.05 ^b,c^	8.24 ± 0.19 ^b,c^
Wet mix-WPI-AHT	64.38 ± 0.78 ^b^	70.57 ± 0.08 ^f^	78.52 ± 0.53 ^c,d^	6.65 ± 0.38 ^d,e^
Dry mix-SC-BHT	66.12 ± 0.38 ^a^	72.97 ± 0.31 ^a,b^	79.26 ± 0.61 ^b,c^	8.73 ± 0.18 ^a,b^
Dry mix-SC-AHT	66.57 ± 0.22 ^2a^	72.51 ± 0.14 ^a,b,c^	79.42 ± 0.35 ^b,c^	8.00 ± 0.06 ^c^
Wet mix-SC-BHT	65.48 ± 0.67 ^a,b^	71.65 ± 0.24 ^c,d^	79.78 ± 0.08 ^b,c^	7.23 ± 0.04 ^c,d^
Wet mix-SC-AHT	64.83 ± 0.40 ^a,b^	70.69 ± 0.32 ^d,e^	78.95 ± 0.11 ^b,c,d^	6.87 ± 0.19 ^d^
Dry mix-G-BHT	66.09 ± 0.42 ^a^	71.21 ± 0.53 ^e^	78.34 ± 0.32 ^c,d^	7.10 ± 0.53 ^d^
Dry mix-G-BHT	65.75 ± 0.30 ^a,b^	72.91 ± 0.28 ^a,b^	79.76 ± 0.11 ^b,c^	6.69 ± 0.55 ^d,e^
Wet mix-G-BHT	66.55 ± 0.71 ^a^	72.05 ± 0.61 ^b,c^	79.08 ± 0.25 ^b,c,d^	7.35 ± 0.28 ^c,d^
Wet mix-G-AHT	65.06 ± 0.20 ^a,b^	70.52 ± 0.05 ^f^	77.87 ± 0.69 ^d^	6.88 ± 0.04 ^d^

WPI: whey protein isolates; SC: sodium caseinate; G: gelatin, BHT: before dry heat treatment; AHT: after dry heat treatment. The different letters in superscript within a column indicate statistically significant differences (*p* < 0.05) within the group.

**Table 2:** (**a**) Texture properties of the corn starch with high amylose (HACS) control and protein-modified HACS before and after DHT. (**b**) Texture properties of waxy corn starch (WCS) control and protein-modified waxy corn starch before and after DHT.

(a)			
Samples	Maximum Force (g)	Cohesiveness	Gumminess
HACS	201.1 ± 3.2 ^a^	0.96 ± 0.05 ^d^	180.32 ± 2.12 ^a^
Dry mix-WPI-BHT	184.0 ± 8.5 ^a^	0.95 ± 0.00 ^a,b^	175.59 ± 8.02 ^b^
Dry mix-WPI-AHT	164.0 ± 1.0 ^a,b^	0.94 ± 0.03 ^a,b^	154.39 ± 0.91 ^c^
Wet mix-WPI-BHT	136.1 ± 4.9 ^a,b,c^	0.94 ± 0.06 ^b,c^	128.57 ± 3.94 ^e^
Wet mix-WPI-AHT	102.8 ± 1.7 ^c^	0.94 ± 0.02 ^b,c^	96.29 ± 0.06 ^g^
Dry mix-SC-BHT	177.3 ± 9.7 ^b,c^	0.95 ± 0.00 ^a,b^	168.52 ± 8.67 ^b^
Dry mix-SC-BHT	146.7 ± 0.2 ^a,b,c^	0.95 ± 0.02 ^a,b^	137.96 ± 0.03 ^d^
Wet mix-SC-BHT	125.3 ± 3.9 ^b,c^	0.93 ± 0.01^2 c^	116.00± 2.56 ^e,f^
Wet mix-SC-AHT	94.4 ± 3.2 ^c^	0.92 ± 0.00^2 c^	86.69 ± 2.57 ^h^
Dry mix-G-BHT	175.9 ± 16.4 ^a,b^	0.95 ± 0.00 ^a,b^	167.64 ± 15.03 ^b,c^
Dry mix-G-BHT	176.4 ± 5.2 ^a,b^	0.93 ± 0.00 ^c^	164.31 ± 4.59 ^b,c^
Wet mix-G-BHT	144.3 ± 1.8 ^a,b^	0.93 ± 0.01 ^c^	135.25 ± 2.75 ^d^
Wet mix-G-AHT	116.7 ± 4.5 ^b^	0.90 ± 0.01 ^d^	105.47 ± 4.03 ^f,g^
**(b)**			
**Samples**	**Maximum Force (g)**	**Cohesiveness**	**Gumminess**
WCS	305.0 ± 2.1 ^a^	0.96 ± 0.00 ^a^	293.21 ± 3.54 ^a^
Dry mix-WPI-BHT	289.1 ± 4.4 ^b^	0.92 ± 0.00 ^a,b^	285.77 ± 20.61 ^a^
Dry mix-WPI-AHT	212.7 ± 2.7 ^b,c^	0.95 ± 0.00 ^a^	202.11 ± 2.93 ^c,d^
Wet mix-WPI-BHT	253.6 ± 17.9 ^b^	0.95 ± 0.01 ^a^	240.50 ± 17.60 ^b^
Wet mix-WPI-AHT	227.4 ± 9.2 ^b,c^	0.94 ± 0.01 ^a^	213.96 ± 0.33 ^c^
Dry mix-SC-BHT	226.6 ± 7.6 ^b,c^	0.88 ± 0.01 ^a,b,c^	200.37 ± 3.79 ^c,d^
Dry mix-SC-AHT	155.6 ± 15.3 ^c,d^	0.86 ± 0.03 ^b,c^	134.17 ± 7.92 ^f^
Wet mix-SC-BHT	240.9 ± 4.2 ^b^	0.93 ± 0.00 ^a,b^	224.53 ± 4.36 ^b,c^
Wet mix-SC-AHT	198.9 ± 9.5 ^b,c^	0.92 ± 0.04 ^a,b^	183.18 ± 0.57 ^d,e^
Dry mix-G-BHT	131.8 ± 1.5 ^c,d^	0.44 ± 0.02 ^f^	57.88 ± 5.04 ^h^
Dry mix-G-AHT	84.9 ± 7.7 ^e^	0.58 ± 0.00 ^e^	48.97 ± 4.98 ^h^
Wet mix-G-BHT	147.3 ± 5.5 ^c,d^	0.71 ± 0.07 ^d^	104.41 ± 11.65 ^g^
Wet mix-G-AHT	199.7 ± 0.7 ^b,c^	0.82 ± 0.03 ^c^	162.91 ± 5.95 ^e^

WPI: whey protein isolates; SC: sodium caseinate; G: gelatin, Control BHT: dry mix of starches and proteins without dry heating. BHT: before dry heat treatment; AHT: after dry heat treatment. The different letters in superscript within a column indicate statistically significant differences (*p* < 0.05) within the group.

**Table 3:** Particle size distribution of native and protein-coated waxy corn starch (WCS) and high amylose corn starch (HACS) prepared by the wet mixing method before and after DHT.

Samples	D× (10) (μm)	D× (50) (μm)	D× (90) (μm)	D (4,3) (μm)
HACS	9.62 ± 0.53 ^e,f^	14.80 ± 0.00 ^h^	22.40 ± 0.16 ^g^	15.50 ± 0.04 ^h^
HACS-WPI-BHT	9.58 ± 0.02 ^f^	15.70 ± 0.00 ^g,h^	79.80 ± 0.00 ^f^	29.50 ± 0.42 ^g^
HACS-WPI-AHT	9.41 ± 0.03 ^g^	16.50 ± 0.05 ^d^	141.00 ± 3.00 ^d^	42.60 ± 2.08 ^f^
HACS-SC-BHT	10.30 ± 0.08 ^b^	21.20 ± 1.36 ^c^	158.25 ± 7.80 ^c^	55.60 ± 5.73 ^b^
HACS-SC-AHT	9.87 ± 0.06 ^c,d^	19.90 ± 0.39 ^d^	156.00 ± 5.00 ^c,d^	52.90 ± 1.86 ^b,c,d^
HACS-G-BHT	9.94 ± 0.04 ^c^	19.10 ± 0.31 ^e^	158.00 ± 4.35 ^c^	51.50 ± 1.82 ^b,c,d^
HACS-G-AHT	9.52 ± 0.07 ^f,g^	18.10 ± 0.38 ^e,f^	154.75 ± 4.50 ^d^	48.70 ± 2.91 ^c,d,e^
WCS	9.60 ± 0.05 ^e,f^	15.30 ± 0.00 ^g,h^	23.80 ± 0.08 ^g^	16.10 ± 0.04 ^h^
WCS-WPI-BHT	9.73 ± 0.05 ^d,e^	16.60 ± 0.10 ^f,g^	119.00 ± 5.00 ^e^	37.00 ± 1.41 ^f^
WCS-WPI-AHT	9.75 ± 0.03 ^d^	18.10 ± 0.13 ^e^	245.00 ± 9.25 ^a^	76.90 ± 4.83 ^a^
WCS-SC-BHT	11.30 ± 0.13 ^a^	31.00 ± 1.16 ^a^	203.00 ± 1.70 ^b^	78.50 ± 1.43 ^a^
WCS-SC-AHT	10.40 ± 0.00 ^b^	24.20 ± 0.14 ^b^	236.00 ± 2.88 ^a^	83.40 ± 1.82 ^a^
WCS-G-BHT	9.95 ± 0.01 ^c^	19.90 ± 0.11 ^d^	159.00 ± 1.50 ^d^	53.50 ± 0.59 ^b,c^
WCS-G-AHT	9.51 ± 0.03 ^f,g^	18.30 ± 0.21 ^e^	140.00 ± 4.27 ^d,e^	45.90 ± 1.45 ^d,e^

WPI: whey protein isolates; SC: sodium caseinate; G: gelatin, BHT: before dry heat treatment; AHT: after dry heat treatment. The different letters in each column indicate statistically significant differences (*p* < 0.05).

## Data Availability

Additional data are available per request.

## Supplementary Materials

The following supporting information can be downloaded at: https://www.mdpi.com/article/10.3390/foods12061350/s1, Figure S1: Particle size distribution of different types of starch-protein combinations obtained by a Mastersizer.
