# Supplementary material for: Effect of Dry Heating on Some Physicochemical Properties of Protein-Coated High Amylose and Waxy Corn Starch
Source: Foods. 2023 Mar 22;12(6):1350. doi: 10.3390/foods12061350 (PMC10048297; doi:10.3390/foods12061350)
Supplement: Supplementary file 1 [file foods-12-01350-s001.zip › foods-2175028-supplementary.pdf]

RMIT Classification: Trusted

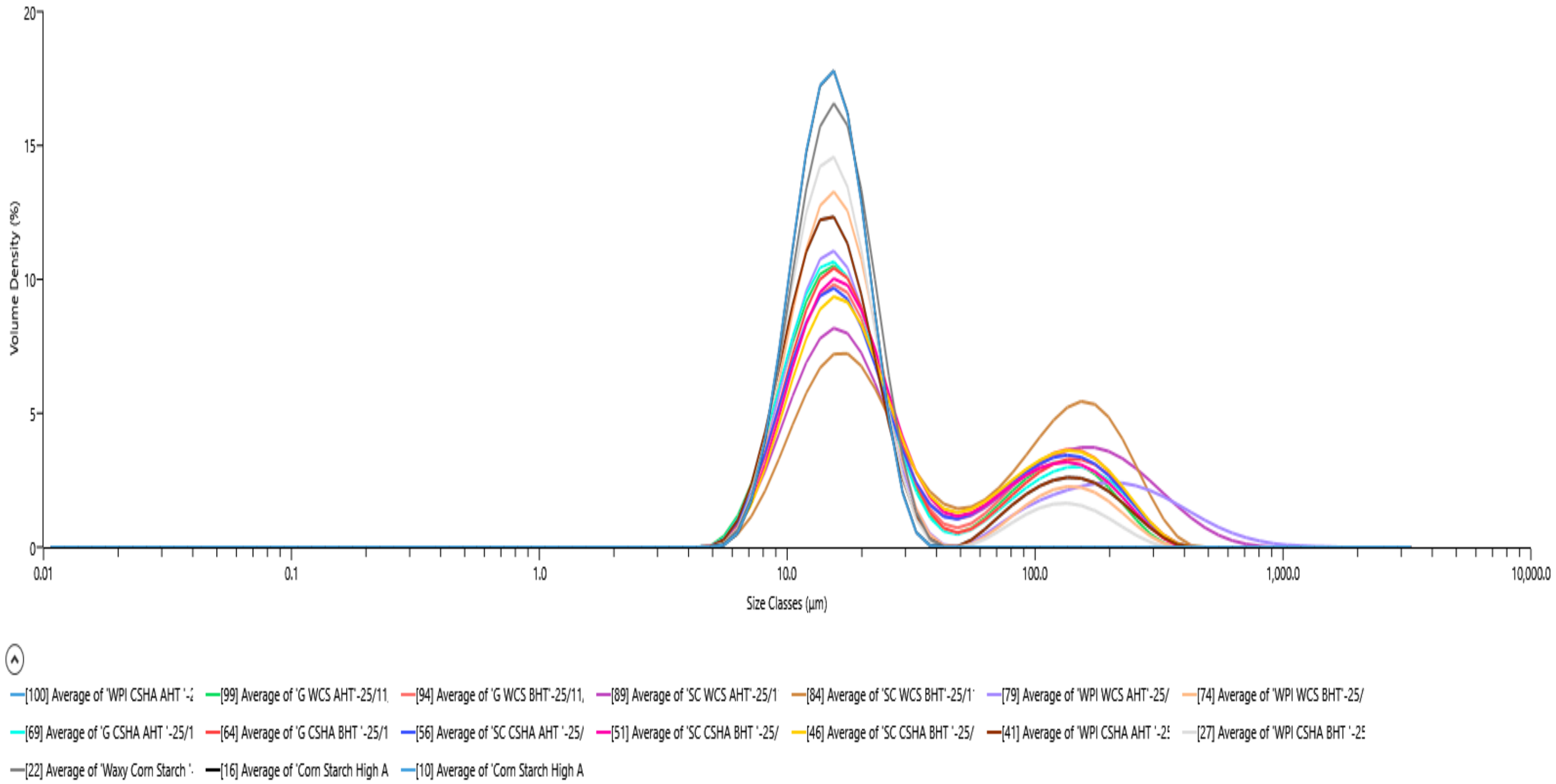

Figure S1. Particle size distribution of different types of starch-protein combinations obtained by a Mastersizer.
